# Prohibitins: A Key Link between Mitochondria and Nervous System Diseases

**DOI:** 10.1155/2022/7494863

**Published:** 2022-07-08

**Authors:** Tianlin Jiang, Jiahua Wang, Chao Li, Guiyun Cao, Xiaohong Wang

**Affiliations:** ^1^School of Medicine, Yangzhou University, Yangzhou 225001, China; ^2^Department of Anesthesia, Affiliated Hospital of Yangzhou University, Yangzhou 225001, China; ^3^Department of Ophthalmology, Linyi People's Hospital, Shandong, China; ^4^Shandong Hongjitang Pharmaceutical Group Co., Ltd., China

## Abstract

Prohibitins (PHBs) are conserved proteins in eukaryotic cells, which are mainly located in the inner mitochondrial membrane (IMM), cell nucleus, and cell membrane. PHBs play crucial roles in various cellular functions, including the cell cycle regulation, tumor suppression, immunoglobulin M receptor binding, and aging. In addition, recent in vitro and in vivo studies have revealed that PHBs are important in nervous system diseases. PHBs can prevent apoptosis, inflammation, mitochondrial dysfunction, and autophagy in neurological disorders through different molecules and pathways, such as OPA-1, PINK1/Parkin, IL6/STAT3, Tau, NO, LC3, and TDP43. Therefore, PHBs show great promise in the protection of neurological disorders. This review summarizes the relevant studies on the relationship between PHBs and neurological disorders and provides an update on the molecular mechanisms of PHBs in nervous system diseases.

## 1. Introduction

**Figure 1:**
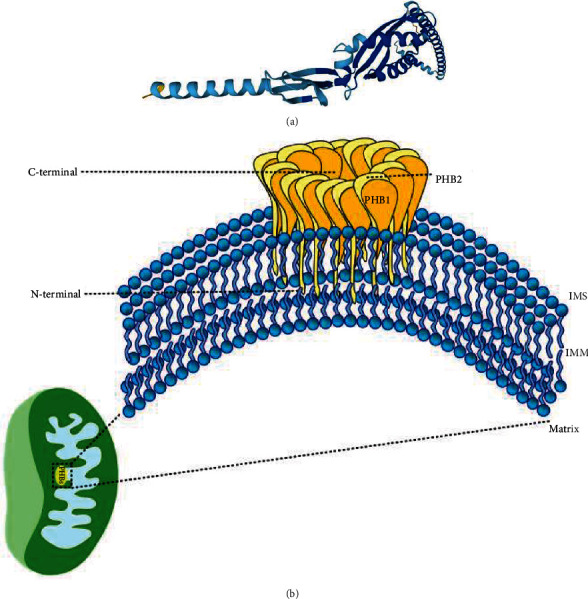
PHBs structures in mitochondrial. (a) The three-dimensional structure of the PHB complex was downloaded from the PubChem database (https://pubchem.ncbi.nlm.nih.gov/), and the human PHB protein in the database was selected as an example to show the microscopic crystal structure of the PHB. (b) The PHB complex is fixed to the IMM by the PHB2 N-terminal domain. The tightly folded C-terminal is exposed to the intermembrane space (IMS).

The first prohibitin (PHB) was discovered in liver tissue by McClung et al. in 1989. This is a highly conserved protein that is widespread in fungi and mammals [1].  Figure 1(a) below reveals the structure of PHB protein in humans.

Members of the PHB family, including PHB1, PHB2, PHB3, and PHB4, were originally found in the cell nucleus and have been identified as inhibitors of cell proliferation [2–4]. The molecular weights of PHB1 and PHB2 are 32 and 37 kDa, respectively [2, 5]. PHB1 and PHB2 both consist of an N-terminal transmembrane domain and a C-terminal coiled-coil domain [2, 4]. The N-terminal domain is evolutionarily conserved, which is similar to other scaffold proteins, and the C-terminal domain is responsible for protein–protein interactions [6, 7]. Steglich et al. suggest that the PHB complex is situated in the inner membrane via its N-terminal transmembrane domains and exposes C-terminal segments into the intermembrane space (IMS) of mitochondria [8]. In mitochondria, as Figure 1(b) shows, PHB1 and PHB2 heterodimers form multimeric ring complexes and bind to newly synthesized products that act as scaffolds to maintain mitochondrial metabolism and structure [9, 10].

Disruption of PHB genes leads to a decreased lifespan, aging, tumors, and metabolic diseases. Postembryonic loss of PHB functions in Caenorhabditis elegans induces pronounced germline defects, such as diminished oocyte production [11–17]. Silencing of PHB in intestinal epithelial cells activates mitochondrial stress-related autophagy, by increasing reactive oxygen species (ROS) levels [18]. Overexpression of PHB resulted in decreased sensitivity to mitochondrial apoptosis and enhanced resistance to various chemotherapy drugs, via downregulation of cytochrome c release in patients with Wilms' tumor [19]. Deletion of PHB2 in the podocytes of mice induced progressive proteinuria, kidney failure, and eventually death [20]. Liver-specific PHB1-knockout in mice caused serious liver injury, including hepatic fibrosis, bile duct epithelial metaplasia, hepatocyte dysplasia, and increased staining for preneoplastic markers [21].

Increasing evidence has shown that PHBs play a vital role in neuronal survival in neurological diseases, including cerebral ischemia, Parkinson's disease (PD), and Alzheimer's disease (AD) by maintaining mitochondrial functions, promoting transcription, and preventing cellular aging [22–26]. However, the role of PHBs is controversial. This review synthesizes knowledge related to the functions of PHB and its molecular mechanisms in the progression of different neurological diseases, providing implications for novel strategies in brain injury.

## 2. Role of PHBs in Mitochondrial Biogenesis

PHB1 and PHB2 form a macromolecular complex at the inner mitochondrial membrane (IMM), with multiple functions, including stabilization of mitochondrial morphology and regulation of mitochondrial apoptosis, mitophagy, fission, and fusion [27]. Depletion or mutations of PHBs directly or indirectly leads to decreased mitochondrial fusion and mitochondrial membrane potential, Ca^2+^ release, increases in ROS levels, and cell death [22].

### 2.1. PHBs Modulate Mitochondrial Dynamics

Mitochondria are highly dynamic organelles that fuse and divide continuously, adjusting their cellular distribution according to the energy demands of the cell. Several mitochondrial proteins are involved in fusion and fission events, including mitofusins 1 and 2 (MFN1, MFN2), optic atrophy 1 protein (OPA-1), dynamin-related protein (DRP1), and fission 1 (FIS1) [28]. Among these, MFN1, MFN2, and OPA-1 are involved in mitochondrial fusion, while DRP1 is required for mitochondrial fission [29]. Without these dynamic processes, the accumulation of fragmented mitochondria induces the clinical manifestation of mitochondrial-related diseases [30].

Activation of DRP1 plays a crucial role in fission. DRP1 interacts with FIS1 to promote the pinching-off and division of mitochondria [31]. Activation of DRP1 is regulated by diverse modifications, including phosphorylation, sumoylation, ubiquitylation, and nitrosylation. Dephosphorylation of DRP1 at S637 induces DRP1 transfer from the cytosol to the mitochondria [32]. Phosphorylation at S616 during mitosis contributes to “mitochondrial replication-like” fission [33]. Inactivation of DRP1 or its partners results in mitochondrial elongation, which has antiapoptotic effects [34]. This has detrimental effects under conditions of cellular division, because elongation greatly interferes with mitochondrial segregation during mitosis [34].

OPA-1 is a crucial protein in mitochondrial fusion, remodeling of the structure of the cristae and apoptosis [35]. OPA-1 can monitor constitutive processes to regulate the “open” and “closed” states of the crista junction [36]. OPA-1 is transformed from an uncleaved long OPA-1 (L-OPA-1) to a cleaved short OPA-1 (S-OPA-1), which blocks cytochrome c release during the initial stage of apoptosis. The PHB complex modulates the dissociation of the cytochrome c complex and the release of cytochrome c, implicating PHB in a key step in apoptosis initiation [37]. Disruption of interactions between mitochondrial-AAA (m-AAA) protease and PHB1 increases cleavage of OPA-1 from the L-OPA-1 to S-OPA-1 forms. PHB2-deficiency accelerates the cleavage of L-OPA-1 to S-isoforms, restoring mitochondrial function and eventually preventing cell apoptosis [38].

PHBs can affect the mitochondrial ultrastructure by interacting with cardiolipin, which is a phospholipid of the IMM [39]. Cardiolipin plays an important role in OPA-1-dependent mitochondrial fusion [40]. After mitochondrial outer membrane fusion, cardiolipin interacts with OPA-1 to fuse the IMM [41]. PHBs play a role in cardiolipin maturation and distribution, functioning as a scaffold and contribute to mitochondrial fusion and restructuring of the mitochondrial cristae [37].

Autophagy occurs in many processes, including development, tumor suppression, and aging [42]. During autophagy, cytosolic constituents are enclosed by a double-membrane vesicle (autophagosome) and are then degraded. Microtubule-associated protein 1 light chain 3 beta (LC3) is an important biomarker of autophagy that participates in the final stages of autophagosome formation [43]. Mitophagy, a different process from autophagy, is a process by which damaged mitochondria are removed [44]. Mitophagy requires parkin to recognize the outer mitochondrial membrane (OMM), which also requires proteasome-mediated degradation of OMM proteins, and then recruits beclin and LC3 [45]. Recently, it was reported that PHB acts as a receptor for selective autophagy of lipidated LC3 (LC3-II) [46].

### 2.2. PHBs Act as Molecular Chaperones for the Stabilization of Mitochondrial Morphology and Functions

PHBs act as molecular chaperones, interacting with other proteins involved in cristae formation, mitochondrial-mediated translation, and maintenance of the mitochondrial structure [9]. The tetrameric complexes of PHB1 and PHB2, formed by interactions of their coiled-coil domains, are anchored in the IMM via the N-terminal hydrophobic regions in PHB1 and PHB2. Thus, PHB1 and PHB2 form a large ring structure in the yeast, *C. elegans*, and in mammals. It has been shown that PHB-knockout induces mitochondrial fragmentation and disorganization in *C. elegans* body-wall muscle cells, mouse embryonic fibroblasts (MEFs), and HeLa cells. Loss of either PHB causes decreased stability of the mitochondrial genome, indicating that the involvement of PHBs is not mandatory for mitochondrial stability [47].

PHB1 and PHB2 complexes can also stabilize mitochondrial protein synthesis and energy metabolism. Silencing or mutation of either PHB1 or PHB2 results in a decrease in mitochondrial membrane potential [48]. PHB1 and PHB2 act as molecular chaperones to assure the expression of mitochondrial transcription factor A (TFAM), which is crucial for mitochondrial DNA replication [49]. A study showed that RNAi-mediated downregulation of PHB1 and PHB2 reduces DNA replication and promotes apoptosis in HeLa cells [49]. In yeast, PHB overexpression can stabilize proteins encoded by the mitochondrial genome and protect newly synthesized proteins from proteases [50]. The C-terminal hydrophobic region of PHB can attach to the newly synthesized polypeptide chain in the mitochondria, assuring its correct folding and stabilizing the mitochondrial structure [51].

PHBs also protect newly synthesized and noncomplexed mitochondrial proteins from being degraded by ATP-dependent m-AAA proteases associated with the large PHB1:PHB2 complexes [52].

Furthermore, PHB2 can stabilize OPA-1 [38], which is a critical target protein of the PHB1/2:m-AAA complex. In PHB2-knockout MEFs, L-OPA-1 was observed to be extremely unstable and rapidly cleaved, which led to arrest of cellular proliferation and increased susceptibility to apoptotic stimuli. Overexpression of a noncleavable L-OPA-1 in PHB2-knockout MEFs completely compensated for PHB2 deficiency, suggesting that the mitochondrial phenotype of PHB2-null MEFs was strictly associated with the proteolytic processing of L-OPA-1 isoforms [53]. Loss of either PHB1 or PHB2 resulted in alterations in the morphology of mitochondrial cristae, in a phenotype similar to the loss of OPA-1 [54].

In nucleoids, PHBs form small nucleoprotein complexes and regulate the stability, replication, and condensation of mitochondrial DNA to stabilize the mitochondrial genome [55].

Berger et al. demonstrated that mutation of PHB1 and PHB2 results in synthetic lethality in trans with mutations of MMM1P (involved in mitochondrial morphology) or MDM10P/MDM12P (involved in mitochondrial DNA maintenance) [56].

### 2.3. Role of PHBs in Maintaining Mitochondrial Respiratory Chain Function

The respiratory complexes consist of the NADH-ubiquinone oxidoreductase (complex I), succinate–ubiquinone oxidoreductase (complex II), ubiquinone–cytochrome c oxidoreductase (complex III), cytochrome c oxidase (complex IV), and the F_1_F_0_–ATP synthase (complex V) [57].

PHBs regulate the stability and translation of respiratory complex proteins by interacting with them in the mitochondria [58]. PHB1 physically interacts with the respiratory chain complex in the IMM, facilitating the assembly and stability of respiratory chain components [59]. In mammals, loss of PHB1 by siRNA treatment contributes to the degradation of complex I and reduces levels of complex IV, while PHB2 overexpression increases mitochondrial complex IV expression, thereby increasing ATP synthesis [7]. PHB2 affects the stability of complex IV by interacting with sphingosine-1-phosphate (S1P). The PHB2:S1P complex binds to cytochrome c oxidase, which is responsible for the correct assembly of the cytochrome c oxidase complex. Loss of PHB2 results in decreased mitochondrial HAX1 and activation of mitochondrial caspase 9/caspase 3, resulting in apoptosis [60].

Expression of PHB is reduced, and mitochondrial oxidative respiratory chain function is impaired in cells treated with tumor necrosis factor-alpha (TNF-*α*) and interferon-gamma (IFN-*γ*), which increases ROS levels [51]. ROS are the by-products of mitochondrial respiration, i.e., electron leakage from respiratory chain complexes I, II, and III. Numerous studies have documented that PHBs are involved in the maintenance of ROS homeostasis [61]. During oxidative stress, PHB1 is exported from the nucleus to the mitochondria. Overexpression of PHB1 downregulates both basal intracellular ROS levels and ROS production, suggesting that PHB1 can protect cells against damage caused by oxidative stress [62]. PHB1 also suppresses ROS levels by increasing the levels of antioxidant enzymes. PHB1 overexpression restores the glutathione S-transferase antioxidant system and activates antioxidant response element (ARE) 4, as well as the expression of antioxidant enzymes NAD(P)H quinone oxidoreductase-1 (NQO-1) and haem-oxygenase-1 (HO-1) [63].

### 2.4. Role of PHBs as Mitochondrial Apoptosis Regulator

Apoptosis is an important process in tissue homeostasis. The release of cytochrome c and the restructuring of mitochondrial cristae are crucial steps in the early stages of mitochondrial apoptosis [64]. In the IMM, the PHB complex plays a key role in maintaining mitochondrial dynamics and function [65]. Overexpression of myocardial PHB1 protects cardiomyocytes from apoptosis induced by hypoxia by inhibiting cytochrome c release and caspase-3 activity, maintaining the mitochondrial transmembrane potential, and increasing levels of Bcl-2 [66]. Mutation of PHB1 and/or PHB2 caused a marked decrease in lifespan and mitochondrial network fragmentation [67]. PHB2 deficiency results in augmented apoptosis in cells, with accumulation of vesicular structures, loss of lamellar-shaped cristae, and impaired mitochondrial dynamics [68].

Importantly, OPA-1 is required for mitochondrial fusion and the integrity of cristae by regulation of the “open” and “closed” states of the crista junction [69]. Oligomerization of L-OPA-1 and S-OPA-1 along the crista junctions blocks the release of cytochrome c and activation of caspase-3 [70]. Loss of PHB2 induces apoptosis by accelerating the cleavage of L-OPA-1 to S-OPA-1 to restore mitochondrial function [71]. This indicates that mitochondrial morphology defects may render cells more susceptible to apoptotic stimulation under PHB2-depletion [72]. The PHB complex, which acts as a lipid scaffold, regulates the dissociation of the cytochrome c/cardiolipin complex and the complete release of free cytochrome c into the cytosol [25]. In addition, it has been speculated that, because of the similar diameters of the PHB complex and cristae tubules, the PHB complex might assemble perpendicularly to the axis of the cristae, stabilizing them and preventing the diffusion of structures within the crista membranes [73].

Moreover, in dysfunctional mitochondria, phosphatase and tensin homolog- (PTEN-) induced putative kinase 1 interacts with parkin, forming a PTEN-induced putative kinase (PINK1)/parkin complex and inducing mitochondrial apoptosis. Han et al. reported that PHB2 inhibits the expression of PINK1 in mitochondria and promotes mitochondrial apoptosis [74, 75].

## 3. Role of PHBs in Inflammation

PHB was shown to play a role in IgM receptor signalling in murine B cells [76]. Many studies have identified the functions of PHB in different immune cell types, such as acting as an adaptor in B cell receptor signalling, play an important role in T cell maturation [77]. In the context of colonic inflammation, PHB overexpression from the VIL1 promoter attenuates inflammation in experimental models of colitis, whereas loss of PHB promotes inflammation during liver injury [78].

The relationship between PHB and immune cells implies that PHB plays an important role in integrating cell signalling with immunometabolism [78]. The PHB1 promoter sequence shows a putative interaction with IL-6, and it also plays a modulatory role in signal transducer and activator of transcription 3 (STAT3) signalling [79]. In nonalcoholic steatohepatitis, IL-6 activates STAT3 and increases PHB1 expression [80]. PHB1 has also been shown to interact with STAT3 to protect cells against TNF-*α*-induced ROS damage to mitochondria [81]. Moreover, IL-6 is associated with the protein level of PHB1 in hepatitis; however, the role of the PHB1/IL-6/STAT3 signalling pathway in the development of hepatitis is controversial [82].

## 4. Role of PHBs in Synaptic Plasticity

Synaptic plasticity is important for hippocampal synapse function, and the plasticity in the hippocampus helps store new memories. Guoyot et al. have shown that treatment with PDD005, which binds PHBs, in aged mice enhances SOX-2 and nestin expression by combining PHBs in CNS cell membranes. Moreover, PDD005 treatment inhibits decline in neuroplasticity. It will be valuable to discover more information on the connection between PHBs and synaptic plasticity because there are only a few studies on this topic.

In addition, unpublished research proves that the level of apoptosis of neuronal cells can be significantly reduced by PHB injection and that the NR2B/CAMK-2/PSD-95 pathway can protect the synaptic plasticity of neurons and, thus, prevent the memory loss phenomenon after cerebral hemorrhage in mice.

## 5. Roles of PHBs in Cerebral Blood Flow

Cerebral blood flow (CBF) is the rate at which arterial blood is delivered to the capillary bed of tissues. It is a measure of nerve function and brain metabolism. Decreased CBF (hypoperfusion) is associated with cognitive impairment, suggesting that cerebrovascular mechanisms are important in maintaining cognitive abilities. NO acts as a vasodilator, and NO donor is involved in telangiectasia [83]. PHBs may protect neurons and regulate cerebral blood flow by binding to NO. This mechanism still needs to be specified. O_2_ can also regulate signal transduction in blood vessels [84]. The production of O_2_ requires mitochondrial action. Ge et al. suggests that PHBs can play a neuroprotective role by increasing blood flow in the brain and thus promoting neuronal regeneration [85]. PHBs also play a role in regulating blood flow after intracerebral hemorrhage (unpublished data). Therefore, we speculate that PHB can indirectly protect cerebral blood flow by protecting mitochondria.

## 6. Protein–Protein Interactions of PHB in Nervous System Disease

Little is known about the subcellular localization and exact function of PHB in the nervous system [52]. In the nervous system, PHB is present not only in neuronal mitochondria; ultrastructural studies have identified the distribution of PHB in postsynaptic dendrites, glial cells, and axon terminals [47]. These results indicate that PHB is involved not only in neuronal mitochondrial function but also in synaptic transmission. To date, many proteins have been identified in complexes with PHBs and play important roles in diverse cellular functions [47]. PHB1 has been shown to colocalize with c-FOS, c-MYC, p53, GnRH, mitochondrial complex I, heterochromatin protein 1 (HP1), ATP10, ATP23, ANT2, MCM2, MCM5, MCM7, and SLP-2 [86]. In the following sections, we discuss major proteins known to interact with PHBs directly and indirectly in neurological and psychiatric diseases.

### 6.1. OPA-1

OPA-1 plays an important role in mitochondrial fusion, remodeling of the crista structure, and apoptosis. Under different stress conditions, OPA-1 is cleaved from L-OPA-1 into S-OPA-1, blocking cytochrome c release [87]. PHB complexes modulate the dissociation of the cytochrome c complex [37]. Disruption of interactions between m-AAA and PHB1 induces an increase in the cleavage of OPA-1 [88]. PHB2 deficiency accelerates cleavage of L-OPA-1 into the S-isoform to restore mitochondrial function [89].

### 6.2. Tau

Tau is predominantly present in neuronal axons, where it binds to and stabilizes microtubules and regulates the axonal transport processes [90]. Hyperphosphorylated forms of tau have been found to detach from microtubules, accumulate in the soma, and aggregate [91]. Phosphorylated tau interferes with the binding of kinesin motors to mitochondria and distinct vesicles, affecting cargo-selective anterograde transport in cultured neurons [92]. Moreover, phosphorylation of tau at AT-8 sites has recently been found to modulate mitochondrial movement in cortical neurons [93]. Tau hyperphosphorylation in the absence of PHB2 causes mitochondrial transport deficiencies, triggering progressive neuronal loss in Phb2 neuronal knockout mice [24].

### 6.3. PINK1

Parkin is recruited to damaged mitochondria by PINK1 to initiate mitophagy [94]. The PINK1–parkin-mediated mitophagy pathway has been widely studied [95]. PINK1 is generally undetectable in healthy mitochondria because it is cleaved by presenilin-associated rhomboid-like (PARL) protein after it is imported into the mitochondrial matrix [96]. Cleaved PINK1 fragments are then released into the cytoplasm where they are degraded by the ubiquitin proteasome system through the N-end rule pathway. Cleaved cytosolic PINK1 fragments inhibit parkin translocation to mitochondria by directly interacting with parkin [97]. However, in damaged depolarized mitochondria, PINK1 is stabilized in the OMM and phosphorylates both ubiquitin and parkin (at Ser65) to activate parkin's E3 ligase activity and recruits parkin from the cytosol to the mitochondria [98].

Yan et al. found an increase in PRKN recruitment to the mitochondria is directly induced by overexpression of PHB2 [75]. Furthermore, PHB2-mediated mitophagy is reliant on the mitochondrial inner membrane protease known as PARL. PARL interacts with PHB2 and becomes active when PHB2 levels drop below a certain threshold. In addition, PGAM5, which is transformed by PARL, contributes to the stability of PINK1 in a manner that is mediated through PHB2.

### 6.4. Nitric Oxide

Nitric oxide (NO), an important signalling molecule, is involved in the control of synaptic functions by modulating neurotransmitter release [99]. NO is involved in the development of ischemic tolerance, termed ischemic preconditioning. PHBs are reported to interact with NO directly, resulting in PHB nitrosylation, which protects cells from neuronal injury [100]. PHB specific mechanisms with NO in CBF still need further proof.

## 7. PHBs Play Important Roles in the Pathology of Nervous System Disease

**Figure 2:**
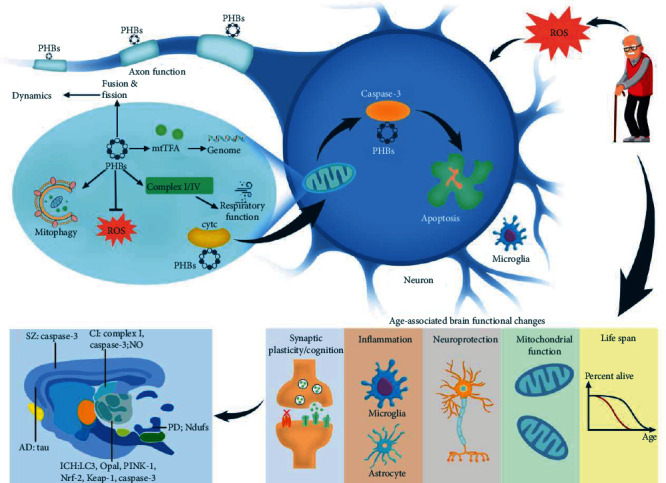
PHB functions in age-related brain functional changes. PHBs play a role in mitochondria through mitochondrial fusion, mitophagy, ROS, and gene synthesis. PHBs in mitochondria enter the cytoplasm of neurons through the combination with cytc, resulting in apoptosis. Moreover, PHBs also appear in axon. PHBs bind to different proteins and participate in the occurrence of different diseases. All these mechanisms contribute to synaptic plasticity/cognition, inflammation, neuroprotection, mitochondrial, and life span.

Figure 2 provides a brief introduction to PHB-specific functions in the nervous system.

### 7.1. PHB in Aging

PHB expression levels in aging cells are significantly lower than those in young cells. Reduced levels of PHBs will lead to mitochondrial defects, resulting in increased lipid levels and abnormal mitochondrial hyperplasia, which further increases ROS production, aggravates cellular aging, and eventually shortens lifespan [75]. The mechanisms by which PHBs are involved in cellular aging may include decreased molecular chaperoning, resulting in an abnormal cytoskeleton and/or decreased maintenance of mitochondria, leading to decreased mitochondrial oxidative respiration [101, 102]. Also, previous study reveals that PHB suppresses aging by protecting synaptic plasticity [101]. All these factors contribute to cellular aging.

### 7.2. PHB in Neurodegenerative Diseases

#### 7.2.1. Parkinson's Disease (PD)

PD is a neurodegenerative disease typically affecting older people, characterized mainly by dopaminergic neuron degeneration and death in the substantia nigra [103]. Mitochondrial dysfunction in PD is characterized by ROS overproduction, loss of ATP and DNA, and glutathione increase [6, 103]. In early PD, impaired mitochondria begin to depolarize and express PINK1 on their outer membrane, which then recruits and activates recombinant parkinsonian disease protein 2 (PARK2), finally leading to ubiquitination and degeneration of OMM proteins. Rupture of the OMM leads to the combination of phagocytic LC3-II and PHB2 in the IMM, resulting in mitophagy [15]. Eventually, the impaired mitochondria are cleared away, alleviating neuronal injury. In MPTP-induced PD models, PHB levels are decreased and mitochondrial injury occurs. PHB loss influences the stability of newly synthesized proteins, eventually impairing mitochondrial function [104]. Conversely, PHB overexpression protects SH-SY5Y cells from MPTP-induced neurotoxicity by strengthening and recovering the activity of NDUFS3, which encodes the 30 kDa subunit of mitochondrial complex I [105].

#### 7.2.2. Alzheimer's Disease (AD)

AD is the foremost neurodegenerative disease in the world, typically affecting people > 65 years old, with a prevalence that increases with age [37, 47]. AD is characterized by tau hyperphosphorylation, amyloid-protein *β* (A*β*) accumulation and mitochondrial autophagy [106]. In healthy people, PHB protects neurons by reducing mitochondrial ROS production [107].

Lachén-Montes et al. found that, in intermediate and advanced AD stages, PHB2 is significantly depleted while phosphorylated isoforms of PHB1 are specifically decreased. Moreover, olfactory PHB expression is also dysregulated in various types of dementia. A mitochondrial imbalance is further seen with upregulation of PHB2 in mixed dementia and downregulation of PHB1 in frontotemporal lobar degeneration [24].

As a molecular chaperone, PHB2 is involved in maintaining the mitochondrial respiratory chain; therefore, its decrease would eventually lead to mitophagy [106]. Aside from reduced respiratory chain activity, autophagosome formation is increased in the brains of patients with AD. In sporadic AD, about 20–50% of patients have TDP-43 lesions. Lower TDP-43 levels decrease PHB2, resulting in mitophagy [24].

#### 7.2.3. Amyotrophic Lateral Sclerosis and Multiple Sclerosis

PHBs also influence other neurological diseases such as amyotrophic lateral sclerosis (ALS) and multiple sclerosis (MS). ALS is a neurodegenerative disease derived from the combined degeneration of upper and lower motor neurons in the spinal cord and motor cortex [22, 108]. PHB levels are significantly reduced in patients with ALS, which is likely related to PHB's interaction with TDP-43 [109].

On the other hand, MS is an autoimmune neurodegenerative disorder mainly caused by oxidative stress and dysfunctional mitochondria [110]. Increased PHB levels in MS brains indicate its protective function against oxidative stress [111].

#### 7.2.4. PHB in Stroke

*(1) Cerebral Ischemia*. Impairment of mitochondrial energy production caused by ischemia–reperfusion may result in neuronal damage in the brain. Hypoxia leads to failure of mitochondria to produce ATP, the stimulation of intracellular calcium ion levels, and an increase in mitochondrial membrane permeability, resulting in membrane potential imbalance and induction of apoptosis [112]. Moreover, impaired mitochondria are the main cause of ROS production after ischemia, which then damages cells by oxidizing proteins and lipids [112].

Kurinami et al. found that PHBs can protect the hippocampal cornu ammonis 1 area neurons from ischemia and can ameliorate postischemic hippocampal dysfunction. By overexpressing PHB in the mouse hippocampus, cytochrome c release was inhibited, and caspase 3 was activated. Thus, PHB can reduce ROS levels after ischemia, inhibit mitochondrial-induced apoptosis, and ameliorate cell death [23].

Qu et al. demonstrated a novel mechanism in the neuroprotective capacity of PHB. They discovered that PHB and NO interact directly, resulting in S-nitrosylation of PHB at Cys69 [100]. They further confirmed that PHB nitrosylation can preserve neuronal viability under hypoxic stress.

*(2) Intracerebral Hemorrhage*. Intracerebral hemorrhage is a severe cerebrovascular disease with high rates of morbidity and mortality [113]. Cerebral hemorrhage resulted in neuronal cleavage of KEAP1 and LC3-II and downregulation of PHB2 levels [62]. Mitoquinone can improve neurological impairment by upregulating the expression of NRF2 and PHB2 [62]. Conversely, NRF2 is inhibited by downregulating the expression of PHB2 and LC3-II [62]. PHB2 siRNA treatment abolished the therapeutic effects of quinone treatment [62]. PHB2 serves as a receptor for mitophagy, binding to LC3-II via its LC3-interaction domain, resulting in parkin-induced OMM rupture. PHB2 also serves as a downstream protein in the PINK1/Parkin pathway. PINK1, Parkin, and LC3-II levels were enhanced after cerebral hemorrhage. However, mitophagy was not significant, and PHB2 levels were found to decrease after cerebral hemorrhage. This indicates that mitophagy is not markedly induced in subarachnoid hemorrhage and that the relationship between the PINK1/parkin pathway and PHB2 does not only involve positive regulation. PHB2, normally located in the IMM, is exposed during mitophagy, promoting clearance of damaged mitochondria and promoting the survival of other mitochondria. However, if cells suffer severe damage, mitophagy would not be effective, and mitochondria would inevitably undergo apoptosis. An increase in mitochondrial death leads to a marked decrease in PHB2 [114].

#### 7.2.5. PHB in Mental Disorder

PHBs have repeatedly been found to be involved in the process and development of schizophrenia [115, 116]. PHBs play diverse roles in schizophrenia, including roles in cell cycle control, apoptosis, aging, and mitochondrial oxidative phosphorylation. All functional impairments might be related to the increase in the number of PHB-immunoreactive oligodendrocytes in the nervous system. Kuwamura et al. found that, in rats with severe myelin destruction, intense immunostaining for PHBs coincided with a marked reduction in ATP and a severely reduced mitochondrial membrane potential [117]. This shows that myelin integrity and mitochondrial dysfunction are involved in schizophrenia, and that an increase in PHB-positive oligodendrocyte (s) plays a crucial role in these processes. Some studies have found that elevated PHB expression in oligodendrocyte(s) contributes to mitochondrial dysfunction and white matter abnormalities in schizophrenia, whereas others have found that PHB exerts cell-protective effects by reducing mitochondrial ROS levels [105]. Smalla et al. found that PHB expression was upregulated in both chronic schizophrenia patients and in a rat ketamine model of this disease. PHB is also a synaptic protein that is present in spine synapses and colocalizes with the presynaptic proteins bassoon and ProSAP2/SHANK3 [118]. Higher PHB levels are also present in postsynaptic densities prepared from the cortex, but not the cerebellum, of rats subchronically treated with ketamine. Quantitative immunoblot analysis of postsynaptic density structures from the left dorsolateral prefrontal cortex of patients with chronic schizophrenia showed that PHB protein levels were significantly elevated in patients with schizophrenia as compared to controls [118].

Table 1 shows the PHB-related mechanisms operating in the neurological diseases addressed above, as well as in other disorders, such as spinal cord injury.

## 8. Conclusions

In this review, we have shown the critical roles played by PHBs in the regulation of mitochondria, transcription, and cellular aging. Taken together, the study findings indicate that PHBs can serve as a potential therapeutic target in neurological disorders, although much of the specific mechanisms remains unknown.

The mechanisms of PHBs activity in aging are unclear, particularly which promoters and inhibitors play a regulatory role in this process. Among them, the role of PHBs in synaptic plasticity is worthy of in-depth study. If successful, it will provide new and powerful evidence for revealing the mechanism of action of synaptic plasticity on memory.

In addition, the specific mechanism of PHBs in neurodegenerative diseases needs to be further studied. In the future, we can combine new research methods to systematically study and network the mechanism of PHBs in neurodegenerative diseases.

Research on the role of PHBs in mental illness is also rare, which can be attributed to the complexity of mental illnesses. If there is more research on PHBs and its role in mental disorders in the future, it will be of great help in the treatment of these diseases.

More efforts are needed to characterize PHBs expression under various circumstances, their biological effects, and the signalling pathways involved.

## Figures and Tables

**Table 1:** PHB-involved mechanisms in neurological diseases.

Neurological disease	PHB change	Cell type	Brain area	Mechanism	Overall impact	Species	Reference (s)
Parkinson's disease	↓	Neuron	Substantia nigra	Ndufs3 binding ↓ ROS generation ↓cytochrome c ↓ PARL-PGAM5-PINK1 ↑ PINK1-PRKN/Parkin ↑	Oxidative stress ↓	Human	[75]
	↑	No	Frontal cortex	No	ATP synthase ↑	Human	
Alzheimer's disease	↓	Olfactory neuron	Olfactory bulb	PHB1 dephosphorylation ↑	The activity of multiple transcription factors ↓	Human	[24]
Multiple sclerosis	↑	Activated T cells	Lymphocytes	No	Protect the brain cells from ROS mediated injury	Human	[7]
Cerebral ischemia	↓	Cortical neurons	Hippocampal CA1 neurons	S-nitrosylation ↓	Mitochondrial free radical production ↓	Rat	[100]
Subarachnoid hemorrhage	↓	Cortical neurons	Hippocampal CA1, CA3, DG neurons	Nrf2/Keap1/PHB2 ↓	Oxidative stress ↓	Rat	[62]
Schizophrenia	↑	Oligodendrocyte	Dorsolateral prefrontal white matter	Oxidative stress ↑	Mitochondrial membrane potential ↓; cell cycle ↓	Rat	[118]
Traumatic brain injury	↑	Astrocyte	Cortex	Interaction with OPA1 ↑	Proliferation level of astrocytes ↑	Rat	[89]
Mental disorder	↑	Cells in hippocampal	Hippocampal	No	Glucocorticoids exposure ↑	Rat	[119]
Spinal cord injury	↓	Neurons	Hippocampal CA1	Bcl-2/Bax/caspase-3 ↑; CCAAT enhancer binding protein, homologous protein, chaperone-ucose-regulated protein 78, and X-box protein 1 ↑; phosphatidylinositol-3-kinase (PI3K)/Akt, extracellular signal-regulated kinase (ERK1/2), and nuclear factor-kappaB ↓	Apoptosis ↑; endoplasmic reticulum stress ↑	Rat	[120]
